# Dr. Winslow on the Effect of Solitary Confinement on the Mind

**Published:** 1849-01-01

**Authors:** Forbes Winslow


### Original Communications and Translations.

### EFFECT OF SOLITARY CONFINEMENT ON THE MIND.*

BY FORBES WINSLOW, M.D.

The important question relative to the influence of what is termed "solitary confinement" on the minds of criminals is, at this moment, exciting much public attention. It is a subject of great gravity. It is not my purpose, on this occasion, to enter at length into its consideration. I merely now write to raise a feeble voice against a system of treatment which, in my humble opinion, is fraught with much mischief to the minds of those unfortunately exposed to its pernicious influence.

Were I disposed, I could cite the particulars of many cases of incurable insanity which I could most undoubtedly trace to this cause. The advocates of the solitary system of treating prisoners may have it in their power to adduce instances subversive of my view of the question, and be able to point with exultation to numerous cases of persons who have escaped unscathed from the solitary cell. This proves nothing. A man may expose himself with impunity to the influence of a most virulent contagion; but, because the poison has no effect upon his constitution, it would be most illogical to infer its non-existence. A man naturally with a strongly constituted mind, united to a vigorous body, may for years be confined in a solitary dungeon, without one ray of light beaming upon his solitude, and his mind may give no indications of diminished power. I am ready to admit, that positive insanity may not develope itself as the effect of solitary confinement. The mind may not be so disturbed as to give rise to "derangement" of the intellect. The perceptive faculties, and even the powers of ratiocination, may present little or no symptoms of disease; these may be, and often are, even in cases of protracted solitary imprisonment, capable of a healthy exercise. But it should never be forgotten, that the mind may be seriously injured, without its presenting any evidences of delusion or false perception. The absence of such morbid phenomena is often referred to as demonstrative of the position, that the solitary system of treating prisoners is not destructive to the sanity of the human mind.

Such reasoners take as a test of insanity, the presence of a false

* This letter was addressed to the Editor of the *Times*.

creation either of the mind or the senses, and will admit no man to be insane who does not believe in the reality of ideas, which have no existence except in his imagination. This is not the philosophical, the medical, or the psychological test of insanity. If positive delusions of the mind are not engendered by the system of treatment, great impairment of the intellectual powers, often amounting to imbecility, are, in many cases, the inevitable, the natural, and melancholy sequel. *A priori* reasoning must force such convictions on the mind. It is an undeniable axiom in physiology, that the brain is the material organ of the mind; and, without discussing the metaphysical question as to the mind being a principle *per se*, capable of a separate and independent existence, no person formed by education to arrive at just conclusions on a subject, necessarily involving in its consideration scientific details, could, for a moment, hesitate in admitting the truth of the position, that the human mind, during its present state, is entirely dependent for its manifestations on the condition of the material organ or organs with which it is associated. The brain is the physical medium through which the mental powers are developed. Such being the fact, the state of the mind is dependent on the condition of the cerebral apparatus. Any agents, be they physical or moral, directly or indirectly interfering with the natural—the healthy action of the brain, must, as a natural consequence, derange or weaken its functions. To guarantee health of mind, it is an indispensable condition that the brain should be regularly exercised. Occupation is essential to the integrity of the mind. The brain, like the organ of digestion, requires food. Mental assimilation must be progressing, materials must be supplied, otherwise the mind will either prey upon itself, or the brain, for want of a stimulus (the stimuli of ideas), will become deteriorated in its physical condition, producing great debility, perhaps imbecility of mind.

To preserve the intellectual powers in a state of health (setting aside altogether the idea of insanity), they must be subjected to regular exercise. If a person be placed in such a position, that he is excluded from all intercourse with his fellow-men, no attempt being made to call the powers of the mind into operation, the brain will fall into a state of atrophy, and great weakness of mind will result, as the natural physiological consequence. This position is undeniable. Experienced men have frequent opportunities of witnessing cases of "impaired mind" (often the most distressing cases to treat), the effect of the mind (or brain) not being sufficiently exercised. Instances, presenting the following characteristics, are not of uncommon occurrence:—

A man accustomed, from early life, to active mercantile pursuits, accumulates a fortune sufficient to enable him to support his family in affluent circumstances. He retires from his counting-house with the determination of spending the remainder of his days in domestic felicity, free from all the anxieties and annoyances incident to the life of a man engaged in the pursuits of commerce. At the commencement of his new career everything looks promising; he appears a contented man. In a short period, he feels the want of something; the mind is not at ease; he is dissatisfied with his position. He then discovers that his ill-health and disturbed mind are the consequences of an abstraction from his accustomed stimuli. He is advised to return to his counting-house, and to resume his former occupation. He does so, and the mind is soon restored to its healthy equilibrium, and he is again a cheerful and a happy man. The above case (which is not a hypothetical one) will illustrate my position, in reference to exercise of mind being an indispensable condition of mental health. I will leave the supporters of the solitary system to prove how this condition can be complied with, under the painful circumstances in which criminals are placed who are subjected to this mode of punishment.

There is another view of the question, which appears to be entirely overlooked. The fact of a man being a criminal is *primâ facie* evidence, not of his being insane, but of his having, if not a predisposition to mental derangement, at least a very irregular, ill-governed, and, it may be, an unhealthy mind. This irregularity of mental operation—this perversion of the moral principle—is often associated with latent insanity; is frequently but one of the many phases which the minds of those assume who are hereditarily predisposed to mental aberration. A man is not necessarily insane, because he is guilty of an atrocious crime; but the tendency to crime is so repeatedly connected with deranged conditions of the mind, that common humanity would induce us to inquire, whether the criminal offence is not the first overt act of insanity?

A woman suddenly jumps up from the breakfast-table, and endeavours to precipitate herself from the window. She is prevented from doing so. To her family and friends she has given no previous indications of insanity. She was calm, collected, and rational in conversation. Apparently, her ideas were not perverted. She engaged zealously in all the active duties of life; in fact, was considered and treated as a person in the possession of a perfectly sound mind. The attempt on her life was thwarted, but from that moment she gave unequivocal indications of a mind greatly disturbed. She was a furious lunatic. Apparently, the attempt at suicide was the first manifestation of her disorder. Had this poor girl (whose case was under my care) succeeded in destroying herself, a verdict of *felo-de-se* might with justice have been recorded. By parity of reasoning, may not an extremely vicious propensity or act be the commencement or premonitory sign of insanity? I have not the least doubt it is so in many cases. As mental aberration often manifests itself in acts which the law considers criminal—as crime is so frequently associated with derangement of mind, and with a constitution predisposed to insanity, it becomes the sacred duty of the Legislature to protect criminals from being exposed to the influence of agents, known both to generate disorders of the mind, and to develop those affections in persons constitutionally liable to them. The time, I trust, is not very remote when more philosophical, and, as a sequence, more liberal views will be taken of those actions designated criminal, and when, without exhibiting any maudlin sentimentality towards those who violate the conditions which bind society together, we shall, in the spirit of our common Christianity, look with great leniency on the faults and failings of our fellow-men.

---

